# Milrinone role in treatment of septic shock

**DOI:** 10.1186/cc14234

**Published:** 2015-03-16

**Authors:** V Tomicic, L Zouein, J Espinoza, S Ugarte

**Affiliations:** 1 Clinica Indisa, Santiago, Chile

## Introduction

The inotropic agents used in the ICU are dobutamine and milrinone; unfortunately, they have shown significant side effects when used for myocardial depression during septic shock. Our objective is to describe Mn behavior in septic shock.

## Methods

We reviewed 72 clinical records of patients with diagnosis of septic and mixed shock who received Mn through January to December 2013. Demographic, hemodynamic, metabolic and gasometric data were recorded before and after Mn infusion. The PiCCO monitoring system was used. Data were expressed as mean and standard deviation. The statistical analysis used Student's *t *test. *P *< 0.05 was considered significant.

## Results

Seventy-two patients were studied: 36.5% were women, mean and SD of age, APACHE II, mechanical ventilation days and long ICU stay were: 67 ± 16 years, 18.5 ± 5.9 points, 14.9 ± 12.9 and 24.5 ± 21.9 days, respectively. A total 20.3% of the patients received dobutamine. Thirty-nine percent presented mixed shock. Global mortality was 23%. After Mn infusion: cardiac index (CI) increased: 3.1 ± 1 to 3.3 ± 1.1, cardiac rate increased: 82.4 ± 14.4 to 88.3 ± 18 and ScvO_2_ increased: 71.1 ± 10.3 to 76.1 ± 7.3 (*P *< 0.05). PaCO_2_ arteriovenous difference and lactate were reduced: 7.36 ± 3.3 to 6.04 ± 3.6 and 18.7 ± 14.9 to 13.1 ± 9.1 (*P *< 0.05). CVP, MAP, RVSI, VSTI, EVLWI and base excess showed no significant difference. Mn initial average dose was 0.35 ± 0.13. NE before and after Mn infusion showed no significant difference: 0.11 ± 0.20 versus 0.12 ± 0.22.

## Conclusion

Mn optimizes cardiovascular performance in septic shock and mixed shock, without affecting hemodynamic variables and global tissue perfusion. In addition, we observed that the IC, ScvO_2_, PaCO_2 _arteriovenous difference and lactate are related variables.

## References

[B1] Holmes CL Vasoactive drugs for vasodilatatory shcok in ICU Curr Opin Crit Care 2009 15 398 402 19542884 10.1097/MCC.0b013e32832e96ef

[B2] Jentzer JC Pharmacotherapy update on the use of vasopressors and inotropes in the intensive care unit J Cardiovasc Pharmacol Ther in press 10.1177/1074248414559838 25432872

[B3] Deep A Evolution of haemodynamics and outcome of fluid-refractory septic shock in children Intensive Care Med 2013 39 1602 9 23812341 10.1007/s00134-013-3003-z

